# Carbon dioxide assimilation and photosynthetic electron transport of tea leaves under nitrogen deficiency

**DOI:** 10.1186/s40529-016-0152-8

**Published:** 2016-11-17

**Authors:** Zheng-he Lin, Qiu-sheng Zhong, Chang-song Chen, Qi-chun Ruan, Zhi-hui Chen, Xiao-mei You

**Affiliations:** Tea Research Institute, Fujian Academy of Agricultural Sciences, Fuan, 355000 China

**Keywords:** Tea plant, Nitrogen deficiency, CO_2_ assimilation, Chlorophyll fluorescence

## Abstract

**Background:**

Tea plant is famed in humid and sub-humid of tropical regions, sub-tropical regions, and is a leaf-harvested crop. Nitrogen is the most important nutrient for increasing quality of tea leaves. Therefore, large amounts of nitrogen fertilizer are increasingly applied by tea farmers. Appropriate application of nitrogen fertilizer aroused people’s concern. This research of physiological response to N deficiency stress will be helpful for appropriate application of nitrogen fertilizer for tea farmers and elucidate a mechanistic basis for the reductions in carbon dioxide (CO_2_) assimilation.

**Results:**

To elucidate a mechanistic basis for the reductions in carbon dioxide (CO_2_) assimilation under nitrogen (N) deficiency tea leaves, changes in chlorophyll (Chl), carbohydrates, ribulose-1,5-bisphosphate carboxylase/oxygenase (Rubisco) and chlorophyll fluorescence transient were examined together with six N treatment (0, 50, 100, 300, 1200 or 6000 μM N). Root, stem and leaves dry weight (DW) increased as N supply increased from 0 to 300 μM, then remained unchanged. The reductions in CO_2_ assimilation of N-deficient leaves paralleled with high intercellular CO_2_ concentration. Rubisco activity, protein and Chl content increased linearly or curvilinearly over the range of leaf N content examined except unchanged as leaf N from 2.15 to 2.79 g m^−2^. Chlorophyll fluorescence transient from N-deficient leaves displayed a depression at the P-step, accompanied by a new step at about 150 μs (L-step). F_v_/F_m_, RE_o_/ET_o_, ET_o_/ABS, S_m_, ET_o_/CS_o_, PI_abs_, PI_tot, abs_, were decreased in N-deficient leaves but increased DI_o_/CS_o_, DI_o_/RC and DI_o_/ABS. Regressive analysis showed that CO_2_ assimilation decreased linearly or curvilinearly with decreasing initial rubisco, PI_abs_ and Leaf Chl, respectively. Therefore, we concluded the decreased photosynthetic electron transport capacity, leaf chl content and initial rubisco activity are probably the main factors contributing to decreased CO_2_ assimilation under N deficiency.

**Conclusions:**

The decreased photosynthetic electron transport capacity, leaf Chl content and initial rubisco activity are probably the main factors contributing to decreased CO_2_ assimilation under N deficiency.

## Background

Tea [*Camellia sinensis* (L.) O.Kuntze] is an evergreen shrub native to China and cultivated in humid and sub-humid of tropical, sub-tropical, and temperate regions of the world mainly on acid soils (Lin et al. 2009). China is the world’s largest tea-producing country, and its tea plantation area had reached 2.87 million hectare (ha) up to 2015, contributing approximately 50% to the world total (Liang and Shi 2015). In addition, tea is a leaf-harvested crop, and nitrogen is the most important nutrient (Silva et al. 2015) for increasing the content of free amino acids, an index of the quality of tea leaves (Tokuda and Hayatsu 2004; Yao et al. 2015). For improving the yield and quality of tea leaves, therefore, large amounts of nitrogen fertilizer are increasingly applied by tea farmers. For instance, the application rates of nitrogen fertilizer to tea plantations have been as high as 450–1200 kg N ha^−1^ year^−1^, which significantly surpasses the recommended rate of 250–375 kg N ha^−1^ year^−1^ for high tea yields (Tokuda and Hayatsu 2004; Hirono and Nonaka 2012; Fu et al. 2012; Zhu et al. 2014). Not surprisingly, such high nitrogen inputs can easily induce excess residual nitrogen and acidification of soil; both influence the nitrogen cycle of tea fields in which a great deal of nitrogenous gases are produced (Jumadi et al. 2008; Zhu et al. 2014). Despite such use, soil N deficiency remains a major constraint on crop productivity in many developing countries. Therefore, increasing tea plant tolerance to low-N conditions would improve tea production, especially in regions with low soil N levels (Wei et al. 2016).

The yield of leaves and leaf photosynthetic rate are directly linked to plant dry matter production. The extent of photosynthesis during tea plant growing can be affected by many factors, including tea cultivars, altitude, climatic condition, CO_2_ level, soil condition and temperature. Another major factor affecting photosynthesis is the available N level of the soil (Wei et al. 2016; Jaaffar and Gardner 1988). Given the diverse roles that nitrogen plays in plant physiology and development, N deficiency has a crippling effect on plants. N deficiency significantly reduces a plant’s capacity for photosynthesis (Boussadia et al. 2010) by reducing the rates of leaf photosynthesis and new leaf area expansion. Furthermore, N deficiency leads to the degradation of photosynthetic pigments and proteins, and reduced enzyme synthesis in plants (Polesskaya et al. 2004). Therefore, N deficiency leads to changes in the expression levels of proteins, as well as the activity levels of enzymes, which invariably leads to changes in plant metabolism (Wei et al. 2016). For example, N levels affect the post-translational modification of phosphoenolpyruvate carboxylase (PEPCase) (Prinsi et al. 2009). Earlier studies in several other crops have also indicated that N deficiency reduces ribulose bisphosphate carboxylase/oxygenase (Rubisco) activity (Chen and cheng 2003, 2004), as well as reducing the actual amount of Rubisco produced by the plant. In addition, N deficiency impacts overall plant metabolism through wide reprogramming of primary and secondary metabolic pathways (Scheible et al. 2004).

The chlorophyll fluorescence transient has been found to be a sensitive indicator of photosynthetic electron transport processes (Tóth et al. 2007). The transient is considered to be determined by changes in the redox state of primary quinine acceptor (Q_A_) (Lazár 2006; Lin et al. 2009), but at the same time, the transient reflects the reduction of the photosynthetic electron transport chain (Schansker et al. 2005; Lin et al. 2009). The chlorophyll fluorescence transient was applied in numerous studies in crop plants, e.g. to assess the environmental effects in wheat, such as drought (Živčák et al. 2008), high temperature (Brestič et al. 2012), light stress (Kalaji et al. 2012; Živčák et al. 2014). The chlorophyll fluorescence transient were applied several times also in studies dealing with nitrogen deficiency in plants and the effect of poor nitrogen supply on photosystem II (PSII) is recently well described (Lu et al. 2001; Redillas et al. 2011; Li et al. 2012). Although in many of published works the rapid chlorophyll fluorescence is denoted as a useful tool for assessing the physiological effects of nitrogen deficiency on plants, there is still a lack of data on the usefulness of the method in assessment of plant photosynthetic performance in crop trials with different nitrogen supply. Thus, it is not well known how N deficiency affects photosynthetic electron transport in tea plant.

Gaining a more complete mechanistic picture of how plants adapt and respond to low N conditions is important since N plays important roles in growth and physiology. This is especially critical for crops like tea, which serves as one of the most popular beverages worldwide (Khokhar and Magnusdottir 2002; Topuz et al. 2014). In addition, a better understanding of the proteins, CO_2_ assimilation and photosynthetic electron transport that influence responses to low N can improve the utilisation efficiency of N fertilisers and assist in developing better methods to evaluate plant responses to possible deficiencies. In this study, we aimed to determine how N deficiency affects CO_2_ assimilation, Rubisco, non-structural carbohydrates and photosynthetic electron transport in tea leaves to understand the mechanism by which N deficiency leads to a decrease in CO_2_ assimilation.

## Methods

### Plant materials and N treatments

The experiment was completed in 2015 in study plot of Tea Research Institute, Fujian Academy of Agricultural Sciences, by using 9-mouth-old uniform tea (*Camellia sinensis* (L.) O. Kuntze *cv.* Benshan) trees potted in 6 L argil pots that were filled with river sand, 2 seedlings per pot, and cultivated in the natural temperature and light conditions. Nutrient solution was prepared by referring to (Lin et al. 2009), and full-strength nutrient solution contained 3 mmol L^−1^ NH_4_NO_3_, 0.5 mmol L^−1^ Ca(H_2_PO_4_)_2_, 1.0 mmol L^−1^ K_2_SO_4_, 0.5 mmol L^−1^ CaCl_2_, 0.6 mmol L^−1^ MgSO_4_, 46 μmol L^−1^ H_3_BO_3_, 9 μmol L^−1^ MnSO_4_, 9 μmol L^−1^ ZnSO_4_, 2 μmol L^−1^ CuSO_4_, 2.6 μmol L^−1^ Na_2_MoO_4_ and 30 μmol L^−1^ Fe-EDTA. Seven weeks after transplanting, the treatment was applied for 18 weeks: until the end of the experiment, each pot was supplied three times weekly with 500 mL of nutrient solution at a N concentration of 0, 50, 100, 300, 1200 or 6000 μM from NH_4_NO_3_ at pH of 5.0. At the end of the experiment, the fully-expanded (about 7 weeks old) leaves from different replicates and treatments were used for all the measurements. Leaf discs (0.63 cm^2^ in size) were collected at noon under full sun and immediately frozen in liquid N_2_. Samples were stored at −80 °C until they were used for the determination of Chl, Rubisco, carbohydrates, and protein. Special care was taken to ensure that all samples were transferred directly from liquid N_2_ to freezer of −80 °C, at no time were any samples exposed to room temperature.

### Measurements of root, stem and leaf DW

At the end of the experiment, 10 plants per treatment from different pots were harvested. The plants were divided into their separate parts (roots, stems and leaves). The plant material was then dried at 80 °C for 48 h and the DW measured. Specific leaf weight was measured according to Syvertsen et al. (1980).

### Determination of leaf total soluble protein, Chl, and total N

Chl, Chl a and Chl b were assayed according to Lichtenthaler (1987). Briefly, 2 frozen leaf discs were extracted with 8 mL of 80% (v/v) acetone for 24 h in the dark. The extracts were determined using Libra S22 ultraviolet–visible spectrophotometer (Biochrom Ltd., Cambridge, UK). Leaf total soluble protein was extracted with 50 mM Na_2_HPO_4_-KH_2_PO_4_ (pH7.0) and 5% (w/v) insoluble polyvinyl-polypyrrolidone (PVPP), and determined according to Bradford (1976) using bovine serum albumin (BSA) as standard. Total N was measured using a continuous flow auto-analyser (AAIII; SEAL Analytical, Germany).

### Leaf gas exchange measurements

Measurements were made with a Li-6400 portable photosynthesis system (PP systems, Herts, UK) at ambient CO_2_ concentration under a controlled light intensity of 1000 μmol m^−2^ s^−1^ between 9:30 and 11:30 on a clear day. During measurements, leaf temperature and vapor pressure deficit (VPD) were 25.9 ± 1.0 °C and 1.9 ± 0.1 kPa, respectively.

### Leaf Rubisco activity measurements

Two frozen leaf discs from the same leaf were ground with a pre-cooled mortar and pestle in 1 mL of extraction buffer containing 50 mM Hepes–KOH (pH 7.5), 10 mM MgCl_2_, 2 mM ethylenediaminetetraacetic acid (EDTA), 10 mM dithiothreitol (DDT), 1% (v/v) Triton X-100, 5% (w/v) insoluble PVPP, 1% (w/v) BSA, 10% (v/v) glycerol. The extract was centrifuged at 13,000*g* for 60 s in 2 °C, and the supernatant was used immediately for the assay of Rubisco activity. Rubisco activity was determined according to Lin et al. (2009). For initial activity, 50 μL of sample extract was added to a cuvette containing 900 μL of an assay solution, immediately followed by adding 50 μL of 10 mM ribulose-1,5-bisphosphate (RuBP), then mixing well. The change of absorbance at 340 nm was monitored for 40 s. For total activity, 50 μL of 10 mM RuBP was added 15 min later, after 50 μL of sample extract was combined with 900 μL of an assay solution to fully activate all the Rubisco. The assay solution for both initial and total activity measurements, whose final volume was 1 mL, contained 100 mM Hepes–KOH (pH 8.0), 25 mM KHCO_3_, 20 mM MgCl_2_, 3.5 mM ATP, 5 mM phosphocretaine, 5 units NAD-glyceraldehyde-3-phosphate dehydrogenase (NAD-GAPDH), 5 units 3-phosphoglyceric phospokinase (PCK), 17.5 units creatine phosphokinase (CPK), 0.25 mM NADH, 0.5 mM RuBP, and 50 μL sample extract. Rubisco activation state was calculated as the ratio of initial activity to total activity.

### Assay of leaf nonstructural carbohydrates

Sucrose, fructose, glucose and starch were extracted 3 times with 80% (v/v) ethanol at 80 °C and determined according to Chen and Cheng (2003).

### Measurements of leaf chlorophyll fluorescence transients

Chlorophyll fluorescence transient was measured by a Handy Plant Efficiency Analyzer (Handy PEA, Hansatech Instruments Limited, Norfolk, UK) according to Strasser et al. (1995). All the measurements were done with 3 h dark-adapted plants at room temperature. Chlorophyll fluorescence transient was induced by 3400 μmol m^−2^ s^−1^ red light, which was provided by three luminous diodes (peak value of 650 nm), and the light was focused on the leaves, evenly illuminated on the exposed/(diameter of 4 mm) surface. At the beginning 300 μs, data were read per 10 μs. With the slowing fluorescence dynamic signals, the time interval of data reading is prolonged. All determination work was conducted at room temperature 3 h after the plants were adapted to the dark condition, and repeat for every 5–7 (each leaf of the seeding repeats once).

For the following derivative parameters: (1) ${\text{V}}_{\text{J}} = \left( {{\text{F}}_{{ 2 {\text{ms}}}} - {\text{F}}_{\text{o}} } \right)/\left( {{\text{F}}_{\text{m}} - {\text{F}}_{\text{o}} } \right)$ denotes the relative variable fluorescence at point J (2 ms); ${\text{V}}_{\text{I}} = \left( {{\text{F}}_{{ 30 {\text{ms}}}} - {\text{F}}_{\text{o}} } \right)/\left( {{\text{F}}_{\text{m}} - {\text{F}}_{\text{o}} } \right)$ the relative variable fluorescence at point I (30 ms); ${\text{S}}_{\text{m}} = {\text{EC}}_{\text{o}} /{\text{RC}} = {\text{Area}}/\left( {{\text{F}}_{\text{m}} - {\text{F}}_{\text{o}} } \right)$ the general electronic carrier of the reaction center; and ${\text{M}}_{\text{o}} = 4\left( {{\text{F}}_{{ 300 \mu {\text{s}}}} - {\text{F}}_{\text{o}} } \right)/\left( {{\text{F}}_{\text{m}} - {\text{F}}_{\text{o}} } \right)$ the initial slope of chlorophyll fluorescence induction curve; (2) The energy used to capture the electron transfer of the unit reaction center (RC): dissipated energy in the ${\text{RC}}:{\text{ DI}}_{\text{o}} /{\text{RC}} = {\text{ABS}}/{\text{RC}} - {\text{TR}}_{\text{o}} /{\text{RC}}$; (3) the quantum yield and energy distribution ratio parameters: the maximum photochemical efficiency ${\upvarphi }_{\text{Po}} = {\text{TR}}_{\text{o}} /{\text{ABS}} = 1 - {\text{F}}_{\text{o}} /{\text{F}}_{\text{m}} = {\text{F}}_{\text{v}} /{\text{F}}_{\text{m}}$; quantum yield for electron transfer ${\upvarphi }_{\text{Eo}} = {\text{ET}}_{\text{o}} /{\text{ABS}} = {\text{F}}_{\text{v}} /{\text{F}}_{\text{m}} \times \left( { 1 - {\text{V}}_{\text{J}} } \right)$; probability of electron transfer by the captured exciton to the electron transfer chain in exceeding other Q_A_ electron acceptors $\uppsi_{\text{Eo}} = {\text{ET}}_{\text{o}} /{\text{TR}}_{\text{o}} = \left( { 1 - {\text{V}}_{\text{J}} } \right)$; quantum yield ratio used for heat dissipation ${\upvarphi }_{\text{Do}} = {\text{DI}}_{\text{o}} /{\text{ABS}} = 1 - {\upvarphi }_{\text{Po}} = {\text{F}}_{\text{o}} /{\text{F}}_{\text{m}}$; (4) quantum yield of electron transfer in unit area (t = 0) ${\text{ET}}_{\text{o}} /{\text{CS}}_{\text{o}} = {\upvarphi }_{\text{Eo}} \times \left( {{\text{ABS}}/{\text{CS}}_{\text{o}} } \right)$; heat dissipation in unit area (t = 0) ${\text{DI}}_{\text{o}} /{\text{CS}}_{\text{o}} = {\text{ABS}}/{\text{CS}}_{\text{o}} - {\text{TR}}_{\text{o}} /{\text{CS}}_{\text{o}}$; (5) Performance index (PI): that is based on light absorption ${\text{PI}}_{\text{abs}} = {\text{RC}}/{\text{ABS}} \times \left[ {{\upvarphi}_{\text{Po}} /\left( { 1 - {\upvarphi }_{\text{Po}} } \right)} \right] \times \left[ {\uppsi_{\text{Eo}} /\left( { 1 -\uppsi_{\text{Eo}} } \right)} \right]$; (6) Total PI, measuring the performance up to the PSI end electron acceptors ${\text{PI}}_{{{\text{tot}},{\text{ abs}}}} = \left( {{\text{RC}}/{\text{ABS}}} \right) \times \left( {{\upvarphi }_{\text{Po}} /\left( { 1 - {\upvarphi }_{\text{Po}} } \right)} \right) \times \left( {\uppsi_{\text{Eo}} /\left( { 1 -\uppsi_{\text{Eo}} } \right)} \right) \times \left( {\updelta_{\text{Ro}} /\left( { 1 -\updelta_{\text{Ro}} } \right)} \right)$.

### Experimental design and statistical analysis

There were 20 pots trees per treatment in a completely randomized design. Experiments were performed with 5–10 replicates (one tree from different pots per replicate). Differences among treatments were separated by the least significant difference (LSD) test at P < 0.05 level.

## Results

### Leaf N content and plant growth characteristics

**Fig. 1:**
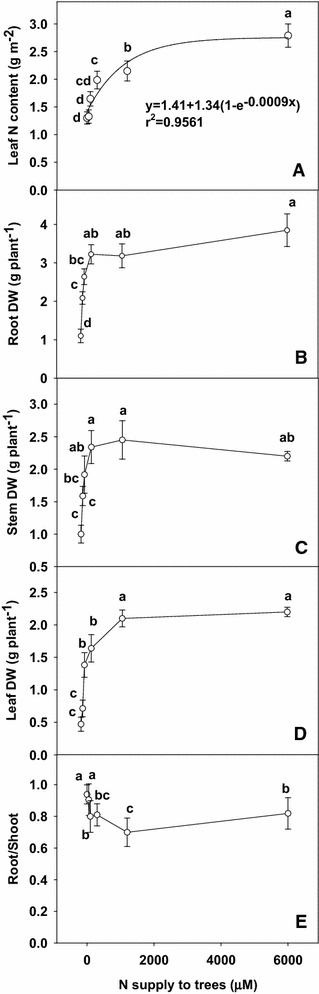
Leaf N content (**A**), root dry weight (**B**), stem dry weight (**C**), leaf dry weight (**D**) and root/shoot dry weight ratio (**E**) of tea trees in relation to nitrogen (N) supply (0, 50, 100, 300, 1200 and 6000 μmol L^−1^). The data were examined using the least significant difference (LSD) test. Each point is mean ± standard error (n = 5). *Different letters* above or below standard *error bars* indicate significant difference at P < 0.05

As N supply decreased, leaf N content decreased curvilinearly (Fig. 1A). Root, stem and leaf dry weight increased as N supply increased from 0 to 300 μM, then remained unchanged (Fig. 1B–D), and resulted in a greater root DW/shoot DW ratio under N supply with 0 and 50 μM (Fig. 1E).

### Leaf Chl, soluble protein, gas exchange and Rubisco

**Fig. 2:**
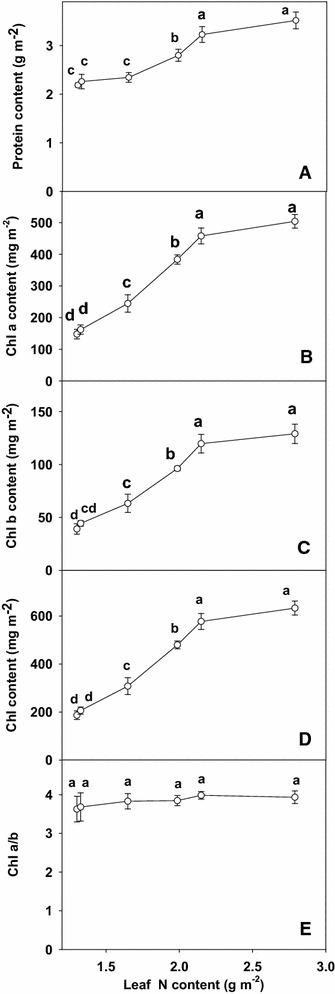
Total soluble protein (**A**), Chl a content (**B**), Chl b content (**C**), Chl content (**D**) and Chl a/b (**E**) in relation to N content (1.30, 1.32, 1.65, 1.99, 2.15 and 2.79 g m^−2^) in tea leaves. The data were examined using a LSD test. Each *point* is mean ± standard error for the leaf N content (*horizontal*, n = 5) and the dependent variable (*vertical*, n = 4). *Different letters* above or below standard *error bars* indicate significant difference at P < 0.05

The total soluble protein contents (Fig. 2A) did not change significantly as leaf N decreased from 2.79 to 2.15 g m^−2^ then decreased significantly with further decreasing leaf N contents. Leaf Chl a (Fig. 2B), Chl b (Fig. 2C) and Chl (Fig. 2D) contents did not change significantly as leaf N decreased from 2.79 to 2.15 g m^−2^ then decreased with further decreasing leaf N content. The ratio of Chl a/b (Fig. 2E) remained unchanged over the range of leaf N content examined.

**Fig. 3:**
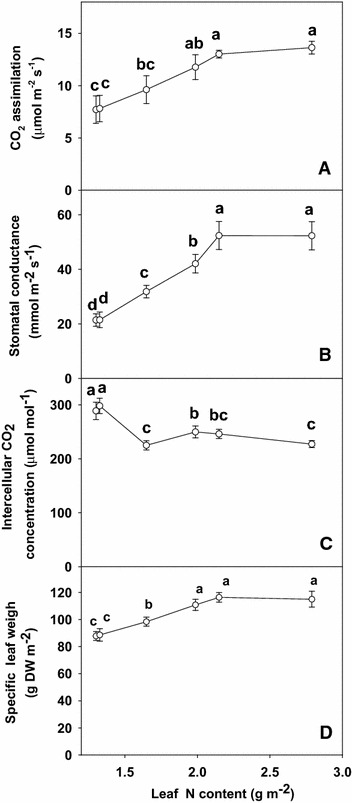
CO_2_ assimilation (**A**), stomatal conductance (**B**), intercellular CO_2_ concentration (**C**) and specific leaf weight (**D**) in relation to N content (1.30, 1.32, 1.65, 1.99, 2.15 and 2.79 g m^−2^) in tea leaves. The data were examined using a LSD test. Each *point* is mean ± standard error for the leaf N content (*horizontal*, n = 5) and the dependent variable (*vertical*, n = 4). *Different letters* above standard *error bars* indicate significant difference at P < 0.05

Leaf CO_2_ assimilation (Fig. 3A) and stomatal conductance (Fig. 3B) increased as leaf N content increased from 1.30 to 2.15 g m^−2^, then remained relatively stable with further increasing leaf N content, whereas intercellular CO_2_ concentration decreased as leaf N content increased from 1.30 to 1.65 g m^−2^, then increased from as leaf N content increased from 1.99 to 2.79 g m^−2^ (Fig. 3C).

**Fig. 4:**
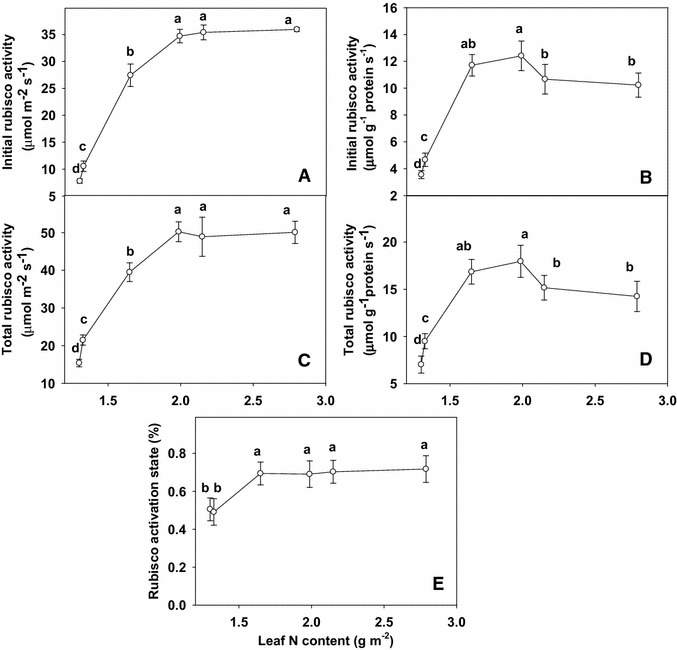
Initial ribulose-1,5-bisphosphate carboxylase/oxygenase (Rubisco) activity (**A**) or expressed on a protein basis (**B**), total Rubisco activity (**C**) or expressed on a protein basis (**D**), and Rubisco activation state (**E**) in relation to N content (1.30, 1.32, 1.65, 1.99, 2.15 and 2.79 g m^−2^) in tea leaves. The data were examined using a LSD test. Each *point* is mean ± standard error for the leaf N content (*horizontal*, n = 5) and the dependent variable (*vertical*, n = 4). *Different letters* above or below standard *error bars* indicate significant difference at P < 0.05

Both initial and total Rubisco activity kept relatively constant as leaf N content decreased from 2.79 to 1.99 g m^−2^, and then decreased significantly with further decreasing leaf N content from 1.99 to 1.30 g m^−2^ (Fig. 4A, C), whereas both initial and total activity expressed on a protein basis did not change significantly, except decreased significantly as leaf N content decreased from 1.33 to 1.30 g m^−2^ (Fig. 4B, D). Rubisco activation state remained unchanged as leaf N content decreased from 2.79 to 1.65 g m^−2^, and then dropped as leaf N content decreased from 1.33 to 1.30 g m^−2^ (Fig. 4E).

### Leaf nonstructural carbohydrates

**Fig. 5:**
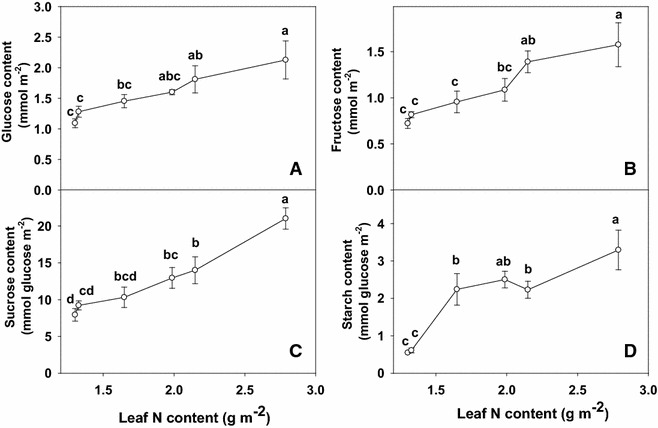
Glucose (**A**), fructose (**B**), sucrose (**C**), and starch (**D**) contents in relation to N content (1.30, 1.32, 1.65, 1.99, 2.15 and 2.79 g m^−2^) in tea leaves. The data were examined using a LSD test. Each *point* is mean ± standard error for the leaf N content (*horizontal*, n = 5) and the dependent variable (*vertical*, n = 4). *Different letters* above standard *error bars* indicate significant difference at P < 0.05

On an area basis, contents of glucose and fructose content did not change significantly as leaf N content decreased from 2.79 to 2.15 g m^−2^ and then dropped significantly as leaf N content decreased from 1.99 to 1.30 g m^−2^ (Fig. 5A, B). The sucrose content increased successive over the range of leaf N content from 1.30 to 2.79 g m^−2^ (Fig. 5C). Leaf starch content remained little changed as leaf N content increased from 1.30 to 1.33 g m^−2^, then increased significantly with further increasing leaf N content (Fig. 5D).

### Leaf chlorophyll fluorescence transients and related parameters

**Fig. 6:**
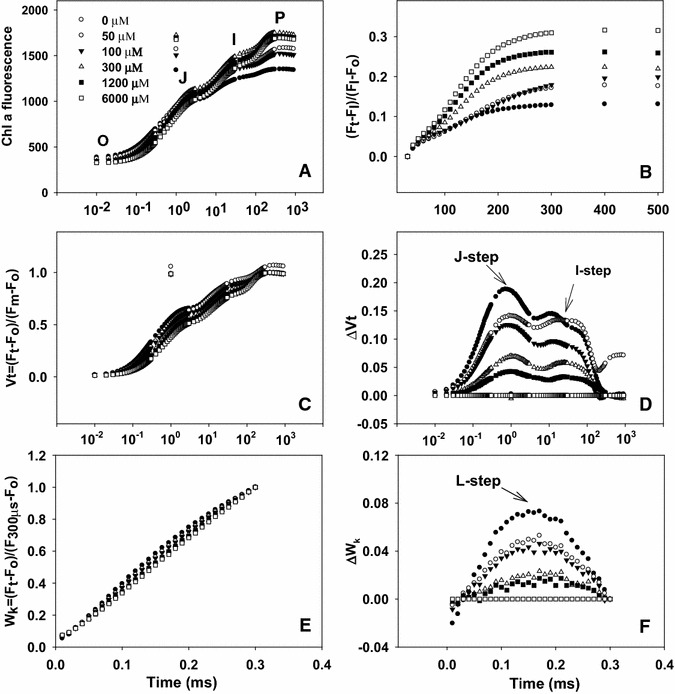
Average chlorophyll fluorescence transients (average of 7–8 samples, **A**) and the different expressions of relative variable fluorescence: (**C**) between F_o_ and F_m_: ${\text{V}}_{\text{t}} = \left( {{\text{F}}_{\text{t}} - {\text{F}}_{\text{o}} } \right)/\left( {{\text{F}}_{\text{m}} - {\text{F}}_{\text{o}} } \right)$ and (**D**) the differences of the six samples to the reference sample treated with 6000 μM N (ΔV_t_), (**E**) between F_o_ and F_300 μs_: ${\text{W}}_{\text{K}} = \left( {{\text{F}}_{\text{t}} - {\text{F}}_{\text{o}} } \right)/\left( {{\text{F}}_{{ 300 \upmu{\text{s}}}} - {\text{F}}_{\text{o}} } \right)$ and **F** the differences of the six samples to the reference sample (ΔW_K_), (**B**) IP phase: $\left( {{\text{F}}_{\text{t}} - {\text{F}}_{\text{o}} } \right)/\left( {{\text{F}}_{\text{I}} - {\text{F}}_{\text{o}} } \right) {-}  1 = \left( {{\text{F}}_{\text{t}} - {\text{F}}_{\text{I}} } \right)/\left( {{\text{F}}_{\text{I}} - {\text{F}}_{\text{o}} } \right)$ in dark-adapted tea leaves under different N supply (0, 50, 100, 300, 1200 and 6000 μmol L^−1^)

**Fig. 7:**
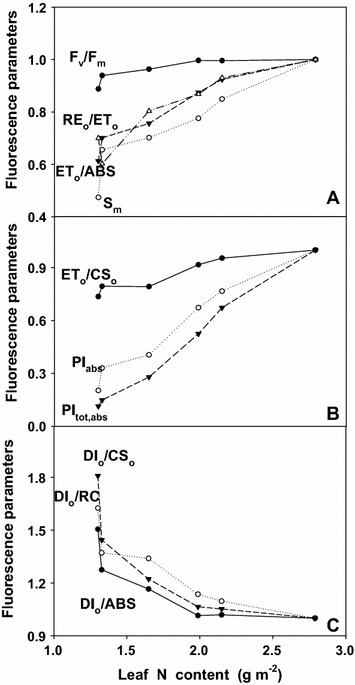
Nine fluorescence parameters (**A** F_v_/F_m_, RE_o_/ET_o_, ET_o_/ABS; **B** ET_o_/CS, PI_abs_, PI_tot_, _abs_; **C** DI_o_/CS_o_, DI_o_/ABS, DI_o_/RC) derived from the average chlorophyll fluorescence transients of Fig. 6A in relation to N content (1.30, 1.32, 1.65, 1.99, 2.15 and 2.79 g m^−2^) in tea leaves. F_v_/F_m_: Maximum quantum yield of primary photochemistry; RE_o_/ET_o_: Efficiency with which an electron can move from the reduced intersystem; ET_o_/ABS: Quantum yield for electron transport; ET_o_/CS:Electron transport flux per CS; PI_abs_: Performance index (PI) on absorption basis; PI_tot_, _abs_: Total PI, measuring the performance up to the PSI end electron acceptors; DI_o_/CS_o_: Dissipated energy flux per CS; DI_o_/ABS: Quantum yield for energy dissipation; DI_o_/RC: Dissipated energy flux per RC. All the values were expressed relative to the sample treated with 6000 μM N set as 1

The chlorophyll fluorescence transients of leaves from 0, 50 and 100 μM N-treated trees showed a large depression at the P-step (Fig. 6A). Figure 6C and D shows the kinetics of relative variable fluorescence at any time ${\text{V}}_{\text{t}} = ({\text{F}}_{\text{t}} - {\text{F}}_{\text{o}} )/({\text{F}}_{\text{m}} - {\text{F}}_{\text{o}} )$ and the differences of normalized N-treated transients minus 6000 μM N-treated transient (ΔV_t_). The differences revealed two obvious bands: increase in the 2–4 ms range J-step and in the 30–100 ms range I-step. The positive J and I-steps were very pronounced in the leaves from 0, 50 and 100 μM N-treated trees. Figure 6E and F depicts the relative variable fluorescence between F_o_ and F_300 μs_ (W_K_) and the differences of normalized N-treated transients minus 6000 μM N-treated transient (ΔW_K_). The differences showed a clear L-step in the leaves from 0, 50 and 100 μM N-treated trees. Figure 6B depicts from 0 to 100 μM N-treated trees had decreased maximum amplitude of IP phase and rise time, and the end-levels were lowered by N deficiency. According to Fig. 7, each parameter the values were normalized on that of the sample treated with 6000 μM N-treated trees. The result showed that leaves N content from 1.30 to 1.65 g m^−2^ had decreased F_v_/F_m_, RE_o_/ET_o_, ET_o_/ABS, S_m_ (Fig. 7A), ET_o_/CS_o_, PI_abs_, PI_tot, abs_, (Fig. 7B), but increased DI_o_/CS_o_, DI_o_/RC and DI_o_/ABS (Fig. 7C).

### Leaf PI_abs_, initial rubisco activity and Chl content in relation to CO_2_ assimilation

**Fig. 8:**
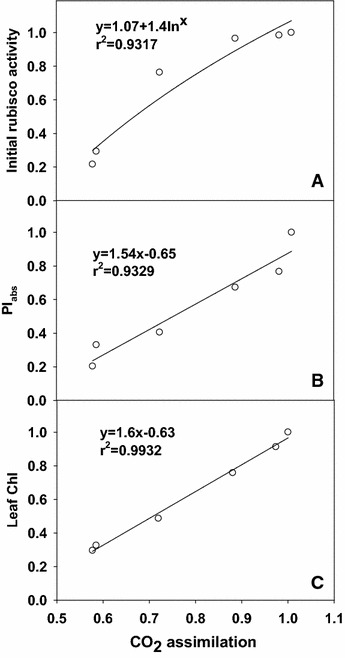
Leaf initial rubisco activity (**A**), PI_abs_ (**B**) and Chl content (**C**) in relation to CO_2_ assimilation in tea leaves. All the values were expressed relative to the sample treated with 6000 μM N set as 1. Regression equations: **A** y = 1.07 + ln^x^ (r^2^ = 0.9317); **B** y = 1.54x − 0.65 (r^2^ = 0.9329); **C** y = 1.6x − 0.63 (r^2^ = 0.9932)

Leaf CO_2_ assimilation increased linearly or curvilinearly with increasing PI_abs_ (Fig. 8A), initial rubisco activity (Fig. 8B) and Chl content (Fig. 8C), respectively.

## Discussion

Nitrogen is one of the most important nutrients for crop growth and development because it affects dry matter production by influencing the leaf area development and maintenance as well as photosynthetic efficiency (Zhu et al. 2014). Nearly all physiological and biochemical activities reached their maximum in the leaves of about 2.15 g m ^−2^ from 300 μM N-treated trees (Figs. 2, 3, 4, 5, 6, 7). Based on these results, trees treated with 0, 50 or 100 μM N are considered N deficient.

The present work (Fig. 1), like that of previous workers (Chen and Cheng 2004; Chen et al. 2015; Zhu et al. 2014) indicates that N deficiency suppressed N content, plant growth and DM accumulation. N deficiency resulted in an increase in the ratio of root/shoot dry weight (Fig. 1E) which agrees with the view that plant tops are affected by N deficiency to a greater extent than root systems (Chen et al. 2015).

Leaf Chl a, b and total Chl (Fig. 2A–C) concentration were closely correlated with leaf N level decreased linearly as leaf N concentration decreased, except for leaf N content from 2.79 to 2.15 g m^−2^, indicating that leaf N no longer limited Chl. These results agree with earlier reports in Grape (Chen and Cheng 2004), sorghum (Zhao et al. 2005), apple (Chen and Cheng 2004) and in corn (Zhao et al. 2003).

N limitation significantly reduced both CO_2_ assimilation and stomatal conductance of tea leaves (Fig. 3A, B), but higher intercellular CO_2_ concentration in low N leaves (Fig. 3C). These indicates that the low CO_2_ assimilation under N limitation was caused by non-stomatal factors (Bondada and Syvertsen 2005). Decreases in CO_2_ assimilation accompanied by an increase in intercellular CO_2_ concentration due to N deprivation has also been reported in wheat (*Triticum aestivum* L.) (Evans 1983), citrus (Bondada and Syvertsen 2005) and Grape (Chen and Cheng 2004). However, the decrease of assimilation CO_2_ rate under N deficiency was accompanied by a decrease in the starch accumulation (Fig. 5D), as previously reported for P deficiency of tea (Lin et al. 2009). This indicates that the production, rather that the utilization of photosynthates, is limiting. The results showed that N-deficiency significantly reduced the contents of glucose (Fig. 5A), fructose (Fig. 5B) and sucrose (Fig. 5C). However, N deficiency was probably not the primary factor limiting CO_2_ assimilation, because there was a greater decrease in CO_2_ assimilation than in sugars content. Evidence shows that soluble sugars, specifically hexoses, may repress photosynthetic gene expression, particularly of the nuclear-encoded small sub-unit of Rubisco, thus decreasing Rubisco content and CO_2_ assimilation (Lin et al. 2009, 2010).

Earlier studies in several other crops indicated that N deficiency reduced either Rubisco activity (Heitholt et al. 1991; Chen and cheng 2004) or the amount of the enzyme (Osaki et al. 1993; Chen and cheng 2003).

In our study, initial and total Rubisco activity expressed on an area basis were closely correlated with leaf N level decreased linearly as leaf N concentration decreased, except for leaf N content from 2.79 to 1.99 g m^−2^, indicating that leaf N no longer limited Rubisco activity. Similar result has been obtained for apple (Chen and cheng 2004) and Grape (Chen and cheng 2003). The decrease in CO_2_ assimilation in N-deficient leaves can not be attributed to a decrease in protein contents, because the decrease in leaf total soluble protein (Fig. 2A) contents was much less than CO_2_ assimilation. Similar results have been reported for maize (Wei et al. 2016), sorghum (Zhao et al. 2005), Olive (Boussadia et al. 2010).

It was well documented that the nitrogen nutrition influences the plant photosynthetic capacity (Terashima and Evans 1988; Živčák et al. 2014). Thus, the membrane processes must be balanced to maintain high efficiency in the conversion of energy and to avoid the over-reduction of photosynthetic electron chain in conditions with different nitrogen supply (Tóth et al. 2007). The decrease of F_v_/F_m_ in N-deficient leaves was caused by a decrease in F_m_ (Fig. 6A, B), as previously found for corn (Jin et al. 2015), maize (Wei et al. 2016) and wheat (Živčák et al. 2014). The decrease in F_v_/F_m_ under stress is considered to reflect the photo inhibitory damage to PSII complexes (Baker and Eva-Rosenqvist 2004).The J-step, I-step and IP phase of chlorophyll fluorescence transients are correlated with the redox state of Q_A_, the redox state of plastoquinone, and the redox state of end acceptors at PSI electron acceptor side, respectively (Lazár 2006; Schansker et al. 2005). The finding that N-deficient leaves had increased V_J_ and V_I_ (Fig. 6C, D), but decreased maximum amplitude of IP phase (Fig. 6B) suggests that acceptor side of PSII became more reduced under N deficiency, but the acceptor side of PSI become more oxidized. A positive L-step appeared at ca. 150 μs in the chlorophyll fluorescence transients in N-deficient leaves (Fig. 6E). This means that the oxygen evolving complex (OEC) is damaged (Hakala et al. 2005). A positive L-step has also been found in N-deficient cowpea leaves (Strasser et al. 1995).

We found that N deficiency decreased F_v_/F_m_, RE_o_/ET_o_, ET_o_/ABS, S_m_, ET_o_/CS_o_, PI_abs_, PI_tot, abs_ (Fig. 7A, B) and increased DI_o_/CS_o_, DI_o_/RC and DI_o_/abs (Fig. 7C). This means that N-deficient leaves damaged all of the photochemical and non- photochemical redox reactions and had a decreased capacity for electron transport, thus limiting ATP synthesis and RuBP regeneration. Regressive analysis showed that CO_2_ assimilation decreased linearly or curvilinearly with decreasing initial rubisco (Fig. 8A), PI_abs_ (Fig. 8B) and Leaf Chl (Fig. 8C), respectively. Therefore, we concluded the decreased photosynthetic electron transport capacity, leaf chl content and initial rubisco activity are probably the main factors contributing to decreased CO_2_ assimilation under N deficiency.

## Conclusions

Assessing the impact of N deficiency on CO_2_ assimilation and photosynthetic electron transport of tea plant is important to improve the utilisation efficiency of N fertilisers and assist in developing better methods to evaluate plant responses to possible deficiencies. This study demonstrated how N deficiency affects CO_2_ assimilation, Rubisco, non-structural carbohydrates and photosynthetic electron transport in tea leaves to understand the mechanism by which N deficiency leads to a decrease in CO_2_ assimilation. The results indicated that the decreased photosynthetic electron transport capacity, leaf chl content and initial rubisco activity are probably the main factors contributing to decreased CO_2_ assimilation under N deficiency.

## Abbreviations

Rubisco: ribulose-1,5-bisphosphate carboxylase/oxygenase
PSII: photosystem II
PI_abs_: performance index (PI) on absorption basis
N deficiency: nitrogen deficiency
Chl: chlorophyll
OEC: oxygen evolving complex
DW: dry weigh
BSA: bovine serum albumin
RuBP: ribulose-1,5-bisphosphate
